# Mathematical model of blood and interstitial flow and lymph production in the liver

**DOI:** 10.1007/s10237-013-0516-x

**Published:** 2013-08-02

**Authors:** Jennifer H. Siggers, Kritsada Leungchavaphongse, Chong Hang Ho, Rodolfo Repetto

**Affiliations:** 1 Department of Bioengineering, Imperial College London, London, SW7 2AZ UK; 2 Department of Civil, Chemical and Environmental Engineering, University of Genoa, Via Montallegro 1, 16145 Genoa, Italy

**Keywords:** Liver, Hemodynamics, Porous medium, Microcirculation, Interstitium, Lymph

## Abstract

We present a mathematical model of blood and interstitial flow in the liver. The liver is treated as a lattice of hexagonal ‘classic’ lobules, which are assumed to be long enough that end effects may be neglected and a two-dimensional problem considered. Since sinusoids and lymphatic vessels are numerous and small compared to the lobule, we use a homogenized approach, describing the sinusoidal and interstitial spaces as porous media. We model plasma filtration from sinusoids to the interstitium, lymph uptake by lymphatic ducts, and lymph outflow from the liver surface. Our results show that the effect of the liver surface only penetrates a depth of a few lobules’ thickness into the tissue. Thus, we separately consider a single lobule lying sufficiently far from all external boundaries that we may regard it as being in an infinite lattice, and also a model of the region near the liver surface. The model predicts that slightly more lymph is produced by interstitial fluid flowing through the liver surface than that taken up by the lymphatic vessels in the liver and that the non-peritonealized region of the surface of the liver results in the total lymph production (uptake by lymphatics plus fluid crossing surface) being about 5 % more than if the entire surface were covered by the Glisson–peritoneal membrane. Estimates of lymph outflow through the surface of the liver are in good agreement with experimental data. We also study the effect of non-physiological values of the controlling parameters, particularly focusing on the conditions of portal hypertension and ascites. To our knowledge, this is the first attempt to model lymph production in the liver. The model provides clinically relevant information about lymph outflow pathways and predicts the systemic response to pathological variations.

## Introduction

The liver is one of the vital organs in the human body, and it plays a fundamental role in numerous functions, including protein synthesis, metabolism, bile secretion, and detoxification. Diseases of the liver are increasingly prevalent in the West, and they represent the fifth most common cause of death in Europe. There are many possible causes of liver disease, including alcohol, viruses, and drugs.

The liver has a circulatory system specific to its function. It is supplied by two major blood vessels: the hepatic artery, which contains fully oxygenated blood, and the hepatic portal vein, which contains partially deoxygenated blood that is rich in nutrients, since it originates from the intestines. Blood flows out of the liver through the hepatic veins. Within the liver, each of the hepatic artery and hepatic portal vein repeatedly bifurcates into successively smaller vessels forming two trees of vessels. On the microscale, the terminal generation of the trees of the hepatic artery and the hepatic portal vein lies, together with bile ducts, in structures called portal tracts. From the portal tracts, blood flows into the sinusoids, a network of small, tortuous, interconnected vessels that carry blood to the central vein, the terminal generation of the hepatic venous tree of vessels. Through successive confluences, blood is carried to the hepatic veins that drain into the inferior vena cava. There are typically around three to seven portal tracts supplying each central vein, and each portal tract supplies about three central veins (Teutsch et al. 1999).

The sinusoids are lined by a layer of fenestrated endothelium. Fenestrations are small holes of approximately 100 nm diameter covering 2–3 % of the area (Burt et al. 2006), which allow plasma to pass from the sinusoids to the space of Disse, a region surrounding each of the sinusoids that is filled with interstitial fluid. The flow from the sinusoids to the interstitial space is driven by both mechanical and oncotic pressure differences between the two spaces. The oncotic pressure difference arises due to proteins in the plasma, but it is normally small compared to the mechanical pressure differences (Laine et al. 1979). The rate of flow from the sinusoids to the interstitium is given by the hepatic filtration coefficient multiplied by the total pressure difference (mechanical plus oncotic) between sinusoids and interstitium. An estimate of this coefficient for cats was found by Greenway et al. (1969).

**Fig. 1:**
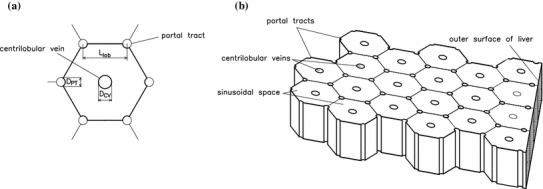
**a** Sketch of a cross section of a single lobule, showing relevant geometrical parameters. **b** Sketch illustrating the arrangement of lobules in the model liver. A section of the outer surface of the model liver is also shown. The surface is assumed to be flat and the axes of the portal tracts parallel to the surface. The surface cuts the lobules so that the outermost lobules have area equal to the interior lobules, although they are pentagonal, rather than hexagonal, as shown. With this arrangement, the outer surface of the liver is at a distance $L_\mathrm{lob}/4$ from the nearest portal tracts and $3L_\mathrm{lob}/4$ from the nearest central veins

On the microscale, the liver can be visualized as being composed of functional units called lobules (Vollmar and Menger 2009). The classic model of a lobule is a prism with a hexagonal cross section, a cylindrical central vein running along the central axis of the prism, and portal tracts along each of the six axial edges (see Fig. 1). The boundaries between lobules are called vascular septa; in some species, such as the pig, these are quite distinct, while in humans the distinction between lobules is less clear (Lautt 2010).

Interstitial fluid is removed from the liver via one of two pathways. The first is through the lymphatic ducts within the liver. There are lymphatic vessels distributed throughout the lobule, and these take up interstitial fluid actively at a regulated rate; however, the dependence of the rate of uptake upon the interstitial pressure and other parameters is not fully known. Elk et al. (1988) performed experiments on livers of anesthetized dogs to determine typical rates of uptake by the lymphatic vessels. In their experiments, they determined the effective resistance of the lymphatic vessels, that is, the increase in interstitial pressure required to produce a unit increase in volumetric flux taken up by the lymphatics. The lymphatic vessels have valves to prevent backflow, and they transport the fluid toward the main lymphatic vessels located in the portal tracts, from where the fluid flows out of the liver. The fluid eventually drains into the venous system at the junction of the left subclavian vein and left jugular vein.

Secondly, interstitial fluid can leave the liver by passing directly through its surface. Conditions of high intrahepatic pressure lead to a pressure imbalance across the surface of the liver, which drives more fluid across it. Different regions of the surface have different properties: On the lower surface, a double membrane comprising Glisson’s capsule and the peritoneal membrane separates the liver from the peritoneal cavity, while the upper surface of the liver is not peritonealized, and there is a space between the liver and the diaphragm in which interstitial fluid can collect. Flow across the liver surface is of particular interest in this paper, because if the flow of interstitial fluid into the peritoneal cavity is too large, fluid can build up in the cavity, leading to a condition called ascites. Ascites, in turn, causes the peritoneal pressure to rise; for example, Laine et al. (1979) performed experiments on anesthetized dogs and found that for every 9.5 ml per kg body weight added to the peritoneum, there is a 1 mmHg rise in the pressure there.

In this paper, we investigate the effect of changes in blood pressure within the liver on the production of lymph by the liver. Such changes are common in small-for-size liver syndrome, which occurs when the functioning liver mass is too small relative to the patient’s body weight and is a relatively frequent complication after partial resection of the liver, after a liver transplantation when the donor is smaller than the recipient, or after living-donor liver transplantation, in both donor and recipient.

There are some previous works on mathematical modeling of the hemodynamics in the liver. Rani et al. (2006) developed a computational fluid dynamics model of flow along a terminal portal vein, hepatic artery, and two sinusoids with fenestrations. They used a non-Newtonian shear-thinning model for the blood rheology. Van Der Plaats et al. (2004) and Debbaut et al. (2011) used electrical analog models to describe the generations of vessels, finding the pressure and flow in each generation. Hoehme et al. (2010) developed a model to quantify regeneration of the liver after lobular damage.

Since the sinusoids are small, numerous, and interconnected, it is reasonable to describe them as a porous medium, and a few models have used this technique. Ricken et al. (2010) developed a poroelastic model of the liver tissue and combined this with a model of the development of sinusoidal orientation to model remodeling of the liver tissue after injury. Bonfiglio et al. (2010) also considered a porous medium model of a single classic hexagonal lobule and analyzed the effects of anisotropic permeability, non-Newtonian effects, and compliance of the tissue. Debbaut et al. (2012a) used a cast of a liver, combined with a computational fluid mechanical simulation, to find the effective permeability of the sinusoids in different directions through the tissue, while Debbaut et al. (2012b) employed these data to develop a three-dimensional lobular model, which they used to investigate the role of the vascular septa.

**Fig. 2:**
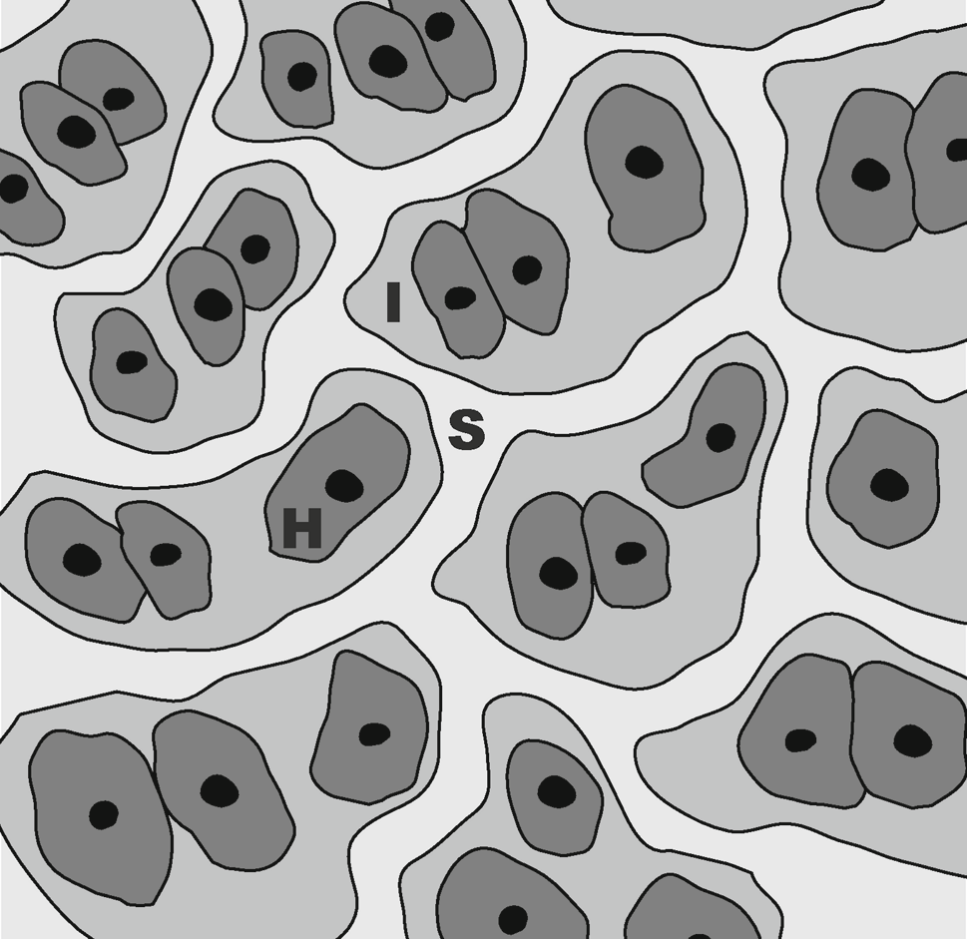
Illustration of modeling assumption concerning the arrangement of cells in the liver at the microscale: ‘H’—hepatocyte (typical diameter 15 $\upmu$m); ‘S’—sinusoid (typical diameter 10 $\upmu$m, Burt et al. 2006); and ‘I’—interstitial space (typical width of space of Disse approximately 500 nm, Straub et al. 2007)

In this paper, we develop a mathematical model of blood and interstitial fluid flow in a lobular model of the liver, in order to estimate the rate of uptake of lymph and the flux of fluid across the surface of the liver. Following Bonfiglio et al. (2010) and Debbaut et al. (2012b), we treat the liver as composed of lobules that are prisms all of equal length, and with no variations in the third dimension. We use a porous medium description of the tissue of the lobules, to describe both the flow in the sinusoids and that in the interstitium. We assume that each spatial point in the model represents a multitude of both sinusoidal vessels and interstitial space, as illustrated in Fig. 2. We prescribe the blood pressure at the portal tracts and central veins, and we assume that blood vessels do not cross the vascular septa from one lobule to its neighbor.

## Mathematical model

### Geometry

In the classic lobule model by Kiernan (1833), each lobule is described as a regular hexagonal prism with portal tracts at each vertex and a central vein along the axis. Since then, this morphological description has been generally accepted as a good idealized representation of the liver lobular structure, and it is reported in many anatomy textbooks. In this paper, we adopt this model and model the entire liver as a lattice of identical hexagonal lobules, each of which has a circular central vein of diameter $D_{CV}$ along its axis and a number of circular portal tracts of diameter $D_{PT}$ at each vertex, as shown in Fig. 1.

We assume that the axial dimension of the lobules is long compared to their width and that the axial pressure gradient is sufficiently small, so that we may treat the flow as two-dimensional in the cross-sectional plane.

The Glissonian–peritoneal membrane is treated as a covering of the lateral faces of the outermost lobules (and not the end faces of the lobules, because end effects are neglected), and similarly to the bare part of the liver surface. We denote the total volume of the liver by $V_\mathrm{liv}$ and its total surface area by $A_\mathrm{liv}$ (estimated in Appendix 6.1) and define $\chi$ as the proportion of the surface area covered by the bare area (the rest being covered by the Glisson–peritoneal membrane). We also assume that the axes of the lobules are parallel to the surface and furthermore assume that the surface cuts the lobules in such a way that the areas of the outermost lobules (now pentagonal prisms) are the same as those of the interior hexagonal lobules, as shown in Fig. 1b.

### Governing equations

Within each lobule, the sinusoids and lymphatic vessels are numerous, and they are small compared to the lobule size. This motivates using a homogenized model for the flow in the sinusoids and in the interstitial space, similar to those considered by Bonfiglio et al. (2010); Debbaut et al. (2012b) and Ricken et al. (2010). We work in terms of the spatially averaged flux per unit area $\mathbf{u}$ instead of the particle velocities $\mathbf{v}$; the spatially averaged flux is the Darcy velocity. In particular, we introduce $\mathbf{u}_S$ and $\mathbf{u}_I$ as the volume-averaged flux per unit area in the sinusoids and in the interstitium, respectively, defined as

1 $$\begin{aligned} \mathbf{u}_S=\dfrac{1}{\varOmega } \int \!\!\int \!\!\int _{\varOmega _S} \mathbf{v} d\varOmega , \quad \mathbf{u}_I=\dfrac{1}{\varOmega } \int \!\!\int \!\!\int _{\varOmega _I} \mathbf{v} d\varOmega . \end{aligned}$$

In the above expressions, $\varOmega$ is an elementary volume, which is significantly larger than the microscale (see Fig. 2) but much smaller than the characteristic scale of a lobule. Moreover, $\varOmega _S$ is the blood volume contained within $\varOmega$ and $\varOmega _I$ the volume of interstitial space in $\varOmega$, so that $\phi _S=\varOmega _S/\varOmega$ and $\phi _I=\varOmega _I/\varOmega$ are the corresponding porosities, with $\phi _S+\phi _I\le 1$. We model the flow using Darcy’s law for flow in a porous medium. Thus,

2 $$\begin{aligned} \mathbf{u}_S=-\frac{k_S}{\mu _S}\varvec{\nabla }p_S,\quad \mathbf{u}_I=-\frac{k_I}{\mu _I}\varvec{\nabla }p_I, \end{aligned}$$

where $p_S$ and $p_I$ are the mechanical pressures of the blood and interstitial fluid, respectively, $k_S$ and $k_I$ are the permeabilities of the sinusoids and interstitial space, respectively, $\mu _S$ is the viscosity of blood, and $\mu _I$ is the viscosity of interstitial fluid. The value of $k_I$ is estimated in Appendix 6.2.

Following Laine et al. (1979), we assume that fluid passes from the sinusoids to the interstitium through the fenestrations in the walls of the endothelial cells at a rate proportional to the pressure difference between the blood and interstitial fluid. The effective pressure difference equals the mechanical pressure difference plus the oncotic pressure difference, but Laine et al. (1979) argue that both the osmotic reflection coefficient and the typical oncotic pressure differences are small, meaning that the flux of plasma from sinusoids to interstitium per unit volume of liver tissue only depends on the mechanical pressure difference and is given by

3 $$\begin{aligned} q_w=C_f\left( p_S-p_I\right) , \end{aligned}$$

where $C_f$ is the hepatic filtration coefficient, equal to the volume flux from the microcirculatory system to the interstitium per unit pressure drop per unit volume of tissue, found experimentally by Greenway et al. (1969) (see also Appendix 6.3).

Within the interstitium, following Elk et al. (1988), we assume that lymph uptake follows a linear relationship

4 $$\begin{aligned} q_l=C_l\max (p_I-p_0,0), \end{aligned}$$

where $C_l$ is the conductance of the lymphatic vessels and $p_0$ is the pressure within the flowing lymph; negative uptake is not possible, due to the presence of valves. See also the papers by Stewart and Laine (2001) and by Quick et al. (2008), and Appendix 6.4.

Applying conservation of mass in both the sinusoids and interstitium, we have

5 $$\begin{aligned} \varvec{\nabla }\cdot \mathbf{u}_S+q_w=0,\quad \varvec{\nabla }\cdot \mathbf{u}_I-q_w+q_l=0. \end{aligned}$$

We can rewrite the system of Eqs. (2)–(5) in terms of the pressures alone as

6 $$\begin{aligned}&-\frac{k_S}{\mu _S}\nabla ^2p_S + C_f(p_S-p_I) = 0,\end{aligned}$$

7 $$\begin{aligned}&-\frac{k_I}{\mu _I}\nabla ^2p_I- C_f(p_S-p_I) + C_l\max (p_I-p_0,0) = 0.\nonumber \\ \end{aligned}$$

### Boundary conditions

We assume that blood does not flow across boundaries between neighboring lobules, due to the presence of the vascular septa, while interstitial fluid flows freely between them, the former condition corresponding to no flux and the latter to continuity of pressure and flux at the boundaries.

At the boundaries of the portal tracts and central veins, and for both the sinusoidal space and the interstitial space, we could choose to prescribe either the pressure or the flux there. In this paper, since there are more relevant data available on the blood pressure, we prescribe the sinusoidal pressures, which are $p_{S,PT}$ at the portal tracts and $p_{S,CV}$ at the central veins. In Sect. 3.3, we argue that reasonable choices of the boundary conditions on the interstitial flow and pressure at the portal tracts and central veins do not significantly affect the results, and in this paper, we assume that there is no flux of interstitial fluid into these vessels.

At the outer surface of the liver, we assume that no blood crosses the surface, corresponding to a no-flux condition, and for the interstitial fluid, we assume that the conductivity of the surface for the interstitial flow equals $M$, and thus,

8 $$\begin{aligned} \mathbf{u}_I\cdot \mathbf{n} = M\left( p_I - p_\mathrm{ext}\right) , \end{aligned}$$

where $p_\mathrm{ext}$ is the pressure external to the liver. The liver surface has two distinct regions with different properties:

- the ‘bare area’ at the upper surface, which has permeability $M=M_{BA}$ and external pressure $p_\mathrm{ext}=p_{DS}$, and
- the lower surface, which is covered by the Glissonian–peritoneal membrane, with permeability $M=M_{GP}$ and external pressure $p_\mathrm{ext}=p_{PC}$.

### Parameter values

A list of the relevant physiological parameters and their typical values is given in Table 1, along with references. These values will be used to produce the results presented in this paper, except where stated otherwise.

**Table 1:** Typical values of physiological parameters taken from the literature

Symbol	Description	Typical value	Ref
$L_\mathrm{lob}$	Typical distance between neighboring portal tracts	500 $\upmu$m	Estimated from Burt et al. (2006) and Lautt (2010)
$D_{PT}$	Diameter of portal tract	50 $\upmu$m	Bonfiglio et al. (2010)
$D_{CV}$	Diameter of central vein	75 $\upmu$m	Bonfiglio et al. (2010)
$V_\mathrm{liv}$	Volume of tissue in liver	1,474 $\hbox {cm}^3$	Wynne et al. (1989) (based on 24-year-olds)
$A_\mathrm{liv}$	Surface area of liver	1,190 $\hbox {cm}^2$	See Appendix 6.1
$\chi$	Proportion of the surface of the liver, that is, bare area	0.2	Estimated from Gray’s Anatomy of the Human Body (1918)
$k_S$	Permeability of sinusoidal space	$1.56\times 10^{-14}\, \hbox {m}^2$	Debbaut et al. (2012a)
$k_I$	Permeability of interstitial space	$0.002\,k_S=3.12\times 10^{-17}\, \hbox {m}^2$	See Appendix 6.2
$\mu _S$	Effective dynamic viscosity of sinusoidal blood	$1.33\mu _I=0.0024$ Pa s	Derived from Eq. (30) in Secomb and Pries (2007), using sinusoidal diameter 10 $\upmu$m (Burt et al. 2006)
$\mu _I$	Dynamic viscosity of interstitial plasma	0.0018 Pa s	Wells and Merrill (1961)
$C_f$	Hepatic filtration coefficient	$5.3\times 10^{-5}$/(mmHg s)	See Appendix 6.3
$C_l$	Lymphatic conductance	$5.9\times 10^{-7}$/(mmHg s)	See Appendix 6.4
$p_0$	Pressure in the flowing lymph	Use 0	
$p_{S,PT}$	Sinusoidal pressure at the portal tracts	4.4 mmHg	Bonfiglio et al. (2010)
$p_{S,CV}$	Sinusoidal pressure at the central veins	1.5 mmHg	Bonfiglio et al. (2010)
$M_{BA}$	Permeability of upper surface of liver	Use $\infty$	
$M_{GP}$	Permeability of Glissonian–peritoneal membrane	$5.7\times 10^{-3} \hbox {ml/h/cmH}_2\hbox {O/cm}^2 =2.15\times 10^{-8}$ m $\hbox {s}^{-1}\hbox {mmHg}^{-1}$	Negrini et al. (1990)
$p_{DS}$	Pressure in diaphragmatic space	Use 0	
$p_{PC}$	Pressure in peritoneal cavity	Use 0	
$Q_\mathrm{blood}$	Flux of blood through the liver	1,717 ml/min $=2.9\times 10^{-5}\,\hbox {m}^3$/s	Wynne et al. (1989) (based on 24-year-olds)
$\gamma$	Fraction of blood entering the liver that is taken up by the lymphatics under normal conditions	$1.0\times 10^{-4}$	See Appendix 6.5

The rows corresponding to $N$ and $L_\mathrm{liv}$ have been deleted

### Numerical computation

We developed a code to simulate the mathematical model using the commercial software COMSOL Multiphysics, which uses a finite element algorithm. The results were validated by successively refining the mesh and checking for convergence, and also comparing against previous data, where possible. In Appendix 5, we also present the analytical solution of a similar problem, in which portal tracts and centrilobular veins are treated as point sources and point sinks, respectively, and their strength is prescribed, rather than the value of the pressure.

The graphical results presented in this paper were plotted using either COMSOL Multiphysics or Matlab.

## Results and discussion

### Single lobule

We first consider the solution for a single lobule in an unbounded lattice of lobules, representing a lobule well into the interior of the liver. Due to the symmetrical setting of the lobule, we apply no-flux boundary conditions on the interstitial flow at each of its straight edges.

**Fig. 3:**
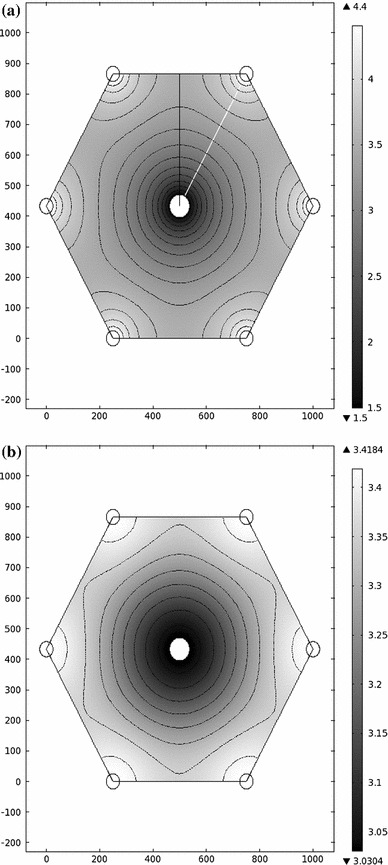
**a** Sinusoidal and **b** interstitial pressures in the model hexagonal lobule (values in mmHg). Contours are spaced by 0.2 mmHg in (**a**) and by 0.05 mmHg in (**b**). Cuts 1 (*black*) and 2 (*white*) are shown in (**a**). Axes in units of $\upmu$m

**Fig. 4:**
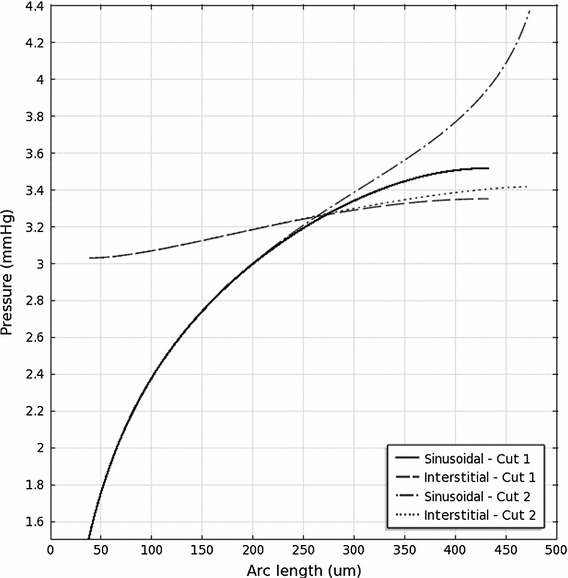
Sinusoidal and interstitial pressures on the cuts shown in Fig. 3

With the parameter values listed in Table 1, the sinusoidal and interstitial pressures are shown in Fig. 3. As expected, the sinusoidal pressure peaks near the portal tracts and is minimized near the central vein. The sinusoidal pressure in the absence of interstitial flow ($C_f=0$), which was studied by Bonfiglio et al. (2010), only differs by about 0.008 % from that obtained in this study (using the same parameter values). The interstitial pressure follows a similar qualitative pattern, but its range is only about 20 % of that of $p_S$. The ranges can be seen in Fig. 4, which shows the pressure on two cut lines through the lobule. The pressure is minimized at the central vein and rises steeply away from this point, which is also where the fastest Darcy velocities are obtained.

**Fig. 5:**
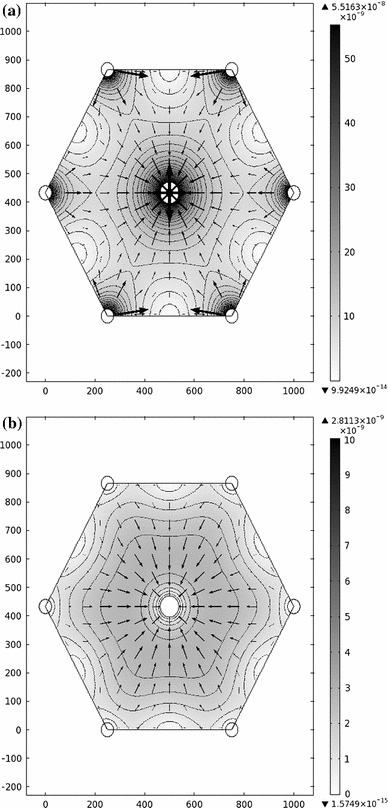
**a** Sinusoidal and **b** interstitial flows in the model (values in m/s). The *shading* and contours show the magnitude of the Darcy velocity (*darker* regions indicate faster flow), and *arrows* indicate direction and magnitude of the flow. The contours in (**a**) are in intervals of $2\times 10^{-9}$ m/s, and those in (**b**) are in intervals of $5\times 10^{-10}$ m/s

Figure 5 shows the magnitudes of the Darcy velocities in the sinusoids and interstitium. In the models by Bonfiglio et al. (2010) and Debbaut et al. (2012b), it was found that the magnitude is maximized near the vessels and minimized at points midway between portal tracts, which is also found in this model. On the other hand, interstitial velocity is minimized near the vessels, due to the boundary conditions imposed there.

The total volume flux of blood into the liver can be estimated using the following formula:

9 $$\begin{aligned} Q_\mathrm{blood}=\frac{V_\mathrm{liv}}{3\sqrt{3}L_\mathrm{lob}^{2}/2}\int -\mathbf{n}\cdot \mathbf{u}_S\,dl, \end{aligned}$$

where $\mathbf{n}$ is the outward-pointing unit normal vector, and the integral is taken around the edge of the lobule. We find this to be approximately $0.66$ l/min, which is around 39 % of the measured physiological value, 1.717 l/min (Wynne et al. 1989). Using the higher value of the permeability, $3.3\times 10^{-13}\,\hbox {m}^2$, estimated by Bonfiglio et al. (2010) (and a proportionately higher value of $k_I$, given by Eq. 37), we find $Q_\mathrm{blood}\approx 14.0$ l/min, about eight times the physiological value, which is almost exactly in proportion to the increase in $k_S$.

It is also of interest to find the rate of fluid taken up by the lymphatics, which equals the average flux per unit volume, $q_l$, integrated over the volume of the liver:

10 $$\begin{aligned} Q_L&= \frac{V_\mathrm{liv}}{3\sqrt{3}L_\mathrm{lob}^{2}/2}\int \limits _{\text {Cross-sectional area}}q_l\,dA\nonumber \\&= \frac{V_\mathrm{liv}}{3\sqrt{3}L_\mathrm{lob}^{2}/2}\int \limits _{\text {Cross-sectional area}}C_l\max (p_I-p_0,0)dA.\nonumber \\ \end{aligned}$$

This gives approximately $0.17$ ml/min, corresponding to about 0.026 % of the total blood volume flux, which is slightly higher than the experimentally derived proportion, $\gamma \approx 0.01\%$, estimated in Appendix 6.5.

The principle of mass conservation implies that there is a relationship between the spatial averages of the pressures, owing to the fact that the flux of blood into the sinusoids minus the flux out equals the net volume flow rate from sinusoids to interstitium equals the rate of uptake of lymph. As long as $p_I>p_0$ everywhere (which is expected to be the case in normal physiological conditions), we have

$$\begin{aligned} \overline{p_I}=\frac{C_f\overline{p_S}+C_lp_0}{C_f+C_l}, \end{aligned}$$

where a bar indicates the spatial average. This equation can also be derived by integrating Eq. (7) over the domain.

### Multiple lobules

**Fig. 6:**
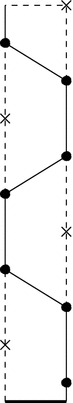
Sketch of the model of the outermost layers of lobules used in numerical simulations to find the behavior in the region near the surface of the liver. Different numbers of lobules were used for different simulations (see, e.g., Fig. 7). *Filled circles* represent the centers of portal tracts, and crosses represent those of central veins. The *thick line* at the *bottom* represents the outer surface of the liver, on which the boundary condition (8) is used. The other *solid lines* represent the boundaries of the lobules, and the *dashed lines* represent lines of symmetry. No-flux conditions are imposed at all the outer edges of the model due to symmetry (except for the *bottom edge*), and the boundary conditions on the interior boundaries are described in Sect. 2.3

Simulations on a lattice consisting of as many lobules as was possible to resolve indicate that the fluid pressure distribution in the lobules that are away from the outer boundary of the model is very close to the pressures in the single lobule simulation that was described in Sect. 3.1. Thus, the effects of the outer surface of the liver seem to be confined to those lobules that are very close to, or bordering, the surface. This suggests that the arrangement of the interior lobules does not significantly influence the rate of interstitial fluid crossing the liver surface; instead, it depends only on the arrangement of the lobules near the outer surface. Thus, in order to estimate the flux across the surface, there is no need to consider a model incorporating the details of the whole liver, and only a model of the near-surface region is required. Therefore, in this section, we consider a simulation of the flow and pressure in a few lobules in a region that borders on the outer surface (see Fig. 6).

**Fig. 7:**
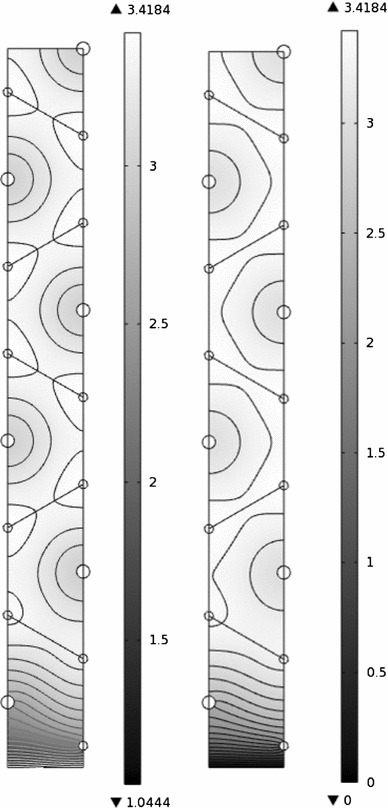
Interstitial pressure in the model near a boundary of the liver (values in mmHg). **a** $M=2.15\times 10^{-8}$ m/s/mmHg, representative of lobules near the Glissonian–peritoneal membrane, and **b** $M=\infty$, representative of lobules near the bare area. The contours are spaced by **a** 0.05 mmHg, **b** 0.1 mmHg

The interstitial pressure in the few outermost lobules at the Glissonian–peritoneal membrane is shown in Fig. 7a. As expected, the interstitial pressure in the innermost lobules in this model is similar to that of the single lobule presented in Sect. 3.1, while the pressure distributions in the two outermost lobules are visibly different, which is due to the boundary conditions imposed at the outer boundary. The range of interstitial pressures in the outermost lobule is about six times that of an internal lobule. Figure 7b shows the interstitial pressure in the outermost lobules near to the bare area. In this case, the effect of the liver surface penetrates through a larger number of lobules than it does near the Glissonian–peritoneal cavity, so a larger number of lobules are needed to resolve the solution. As in Fig. 7a, the pressure distributions in the innermost lobules in Fig. 7b are similar to that in the single lobule solution in Sect. 3.1. The range of interstitial fluid pressure in the outermost lobule at the bare area is about nine times that of the inner lobules.

The fluid loss through the surface of the liver equals the average flux per unit area through the surface multiplied by the surface area. Thus, the flux through the Glissonian–peritoneal membrane equals

11 $$\begin{aligned} Q_{GP}&=\frac{A_{GP}}{L_\mathrm{edge}}\int \limits _\mathrm{edge}\mathbf{u}_I\cdot \mathbf{n}\,dl\nonumber \\&=(1-\chi )\frac{A_\mathrm{liv}}{L_\mathrm{edge}}\int \limits _\mathrm{edge}M_{GP}\left( p_I-p_{PC}\right) \,dl\nonumber \\&\approx 0.14\,\text {ml/min}, \end{aligned}$$

where $A_{GP}\!=\!(1-\chi )A_\mathrm{liv}$ is the area covered by the Glissonian–peritoneal membrane, the integral is taken along the lower edge of the bottom lobule in Fig. 7a, and the numerical value is derived using the parameter values in Table 1. Similarly, the flux through the bare area is given by

12 $$\begin{aligned} Q_{BA}&=\frac{A_{BA}}{L_\mathrm{edge}}\int \limits _\mathrm{edge}\mathbf{u}_I\cdot \mathbf{n}\,dl\nonumber \\&=-\chi \frac{k_IA_\mathrm{liv}}{\mu _IL_\mathrm{edge}}\int \limits _\mathrm{edge}\mathbf{n}\cdot \varvec{\nabla }p_I\,dl\nonumber \\&\approx 0.051\,\text {ml/min}, \end{aligned}$$

where $A_{BA}=\chi A_\mathrm{liv}$ is the bare area, and the integral is taken along the bottom edge in Fig. 7b. The proportion of the space covered by the bare area, $\chi$, is not well known: However, the results presented here are qualitatively independent of its value. The total flux crossing the surface is $Q_\mathrm{surface}=Q_{GP}+Q_{BA}\approx 0.19$ ml/min under normal physiological conditions, which is about 0.028 % of the flux $Q_\mathrm{blood}$ listed in Table 1, and about 1.1 times $Q_L$. This implies that the bare area leads to an increase in the total flux crossing the liver surface by around 10 % compared to the flux that would be obtained if the entire surface were peritonealized.

The total rate of lymph production by the liver equals $Q_\mathrm{liver-lymph}=Q_L+Q_\mathrm{surface}\approx 0.36$ ml/min, which corresponds to 0.51 liters per day of fluid production; this is of the same order of magnitude as the measured physiological values.

### Effect of abnormal physiology and variation in the model parameter values

In this section we consider the effect of changing certain model parameters on the rates of lymph and peritoneal fluid production; we investigate the parameters whose values are uncertain and also parameters that are known to vary in medical conditions of interest.

**Fig. 8:**
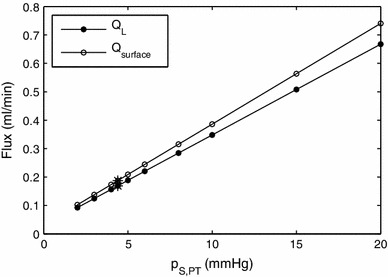
Effect of change in $p_{S,PT}$ on the rates of lymph uptake in the liver and production of peritoneal fluid by the liver. *Asterisks* mark the physiological values

One of the parameters to which the sensitivity of the model is of most interest is the sinusoidal pressure at the portal tracts, because this is known to increase during portal hypertension in small-for-size liver syndrome. As can be seen in Fig. 8, the flows increase linearly with increasing pressure, and the rate is about 0.032 ml/min per mmHg pressure rise for lymphatic uptake and about 0.035 ml/min per mmHg for fluid crossing the liver surface.

**Fig. 9:**
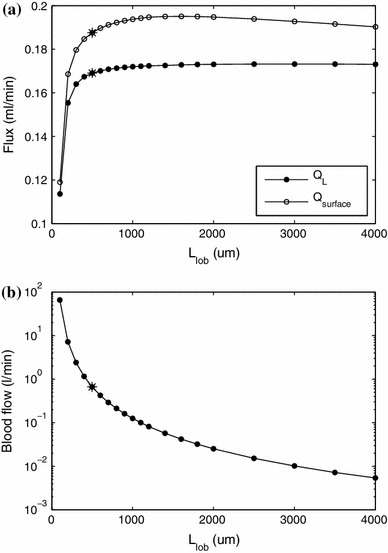
Effect of change of lobule side length on the rates of lymph uptake in the liver and production of peritoneal fluid by the liver (**a**) and blood flux (**b**)

**Fig. 10:**
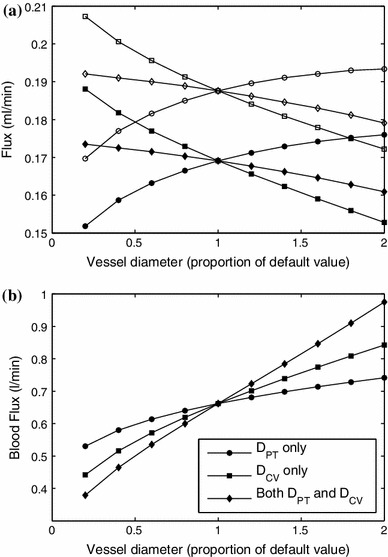
Effect of change of vessel diameter on the rates of **a** lymph uptake and flow across the surface of the liver and **b** flux of blood through the liver. In (**a**) only, *solid symbols*—$Q_L$; *open symbols*—$Q_\mathrm{surface}$. In both (**a**) and (**b**), *circles* denote the effect of changing $D_{PT}$ only, *squares* denote the effect of changing $D_{CV}$ only, and *diamonds* denote the effect of changing both $D_{PT}$ and $D_{CV}$ simultaneously

In Fig. 9 we show the effect of changing the distance between neighboring portal tracts $L_\mathrm{lob}$ on lymph uptake in the liver and production of peritoneal fluid by the liver (a) and on blood flux (b). The first figure shows that the effect of the lobule size on lymph flow is fairly small over a very wide range of values of $L_\mathrm{lob}$ (much wider than physiologically realistic). The effect becomes strong only for unrealistically small values of the size of the lobule. This is because, in this case, the lymph has to flow around the vessels that for small values of $L_\mathrm{lob}$ occupy a large percentage of the whole cross section of the lobule. On the other hand, as expected, blood flux is extremely sensitive to the lobule size, as shown in Fig. 10b.

Since histological images show wide variation in vessel diameters, in Fig. 10a we plot the effect of vessel diameter on the flux. Increasing the size of the portal tracts increases both the uptake of lymph and the production of fluid by the liver. This is because in this case there is less resistance near the portal tracts, meaning the sinusoidal pressure is higher in regions close to them. In turn, this means the interstitial pressure has a higher average value, and thus, both the uptake of lymph (directly related to interstitial pressure via Eq. (4)) and the flux across the surface of the liver (given by (8)) are increased. Increasing the size of the central vein decreases both of these fluxes because there is less resistance, meaning the sinusoidal pressure is lower near it, and thus, the average interstitial pressure is smaller too, leading to both a lower rate of lymphatic uptake and also less fluid crossing the liver surface. Increasing the diameters of both portal tracts and central veins in proportion to one another has relatively little effect on these fluxes because the sinusoidal pressure distribution stays approximately unchanged, and thus, the interstitial pressure is largely unaffected. The flux of blood, shown in Fig. 10b, increases monotonically if the size of any vessel increases, since the vessel’s surface area increases, which decreases the resistance to blood flow. The size of the central vein has a greater effect on the flux than the portal tracts, because there are more portal tracts, so they collectively offer less resistance.

**Fig. 11:**
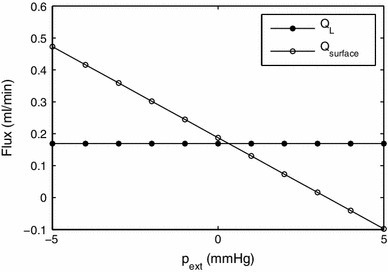
Effect of the pressure external to the liver in the peritoneal cavity, $p_{PC}$, and in the diaphragmatic space, $p_{DS}$ ($p_{PC}=p_{DS}$ is assumed, and these pressures are collectively denoted by $p_\mathrm{ext}$), on the rates of uptake by the lymphatics and flow through the surface of the liver

During ascites, the peritoneal pressure increases. In Fig. 11 we investigate the effect of different values of $p_\mathrm{ext}$. For simplicity, we used $p_{PC}=p_{DS}$ and denote them both by $p_\mathrm{ext}$. In this case, the lymph uptake, $Q_L$, does not change significantly, whereas the outflow from the liver surface, $Q_\mathrm{surface}$, decreases significantly and, according to the model, might even be reversed. Although the mathematical boundary condition (8) does not prevent flow from the abdomen to the liver, we are not aware of any evidence of its possible occurrence.

**Fig. 12:**
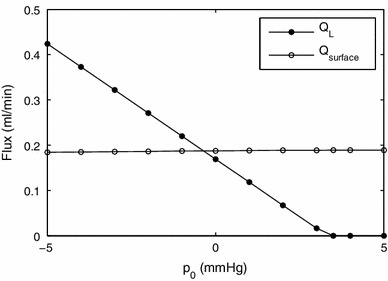
Effect of flowing lymph pressure $p_0$ on the rates of uptake by the lymphatics and flow through the surface of the liver

We also investigated the effect of changing the value of the flowing lymph pressure, $p_0$, as values for this parameter were not found in the literature, which is shown in Fig. 12. Increasing $p_0$ has only a small effect on the outflow from the liver surface, whereas it strongly affects the uptake from lymphatic vessels, which decreases approximately linearly with $p_0$ as $p_0$ increases. For sufficiently large $p_0$, it vanishes, because $p_I<p_0$ everywhere, so no fluid is taken up by the lymphatics. We also note that the effects of $p_\mathrm{ext}$ on $Q_\mathrm{surface}$ and of $p_0$ on $Q_L$ are analogous to one another; however, there is a qualitative difference for high values of these external pressures, which occurs because, for high $p_0$, the valves in the lymphangions prevent backflow, and there is no corresponding mechanics for high $p_\mathrm{ext}$.

**Fig. 13:**
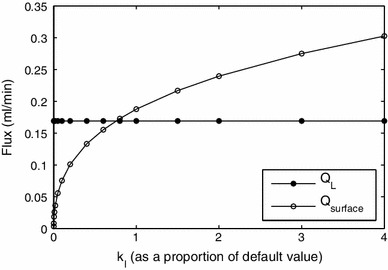
Effect of the value of the permeability of the interstitial space, $k_I$, on the rates of lymph production. For larger values of $k_I$, a larger number of lobules are needed in the model to resolve the simulation; for example, for $k_I$ four times the default value, we used a model with 10 lobules

The permeability of the interstitial space, $k_I$, could not reliably be determined from experimental data, and it is estimated in Appendix 6.2. In Fig. 13 we show how the lymph production depends on $k_I$. The flux through the surface increases as $k_I$ increases, and tends to zero for vanishingly small values of the permeability, while $Q_L$ is unaffected by the value of $k_I$. The increase in $Q_\mathrm{surface}$ is due to a reduction in the resistance of the outflow pathway through the liver surface for higher values of $k_I$.

Since there could be interstitial flow within the portal tracts and central veins, the authors also considered a modified model. In this model, within the portal tracts and central veins, the interstitial flow satisfies

13 $$\begin{aligned} \varvec{\nabla }\cdot \mathbf{u}_I+q_l\!=\!0 \Rightarrow -\frac{k_I}{\mu _I}\nabla ^2p_I\!+\! C_l\max (p_I-p_0,0)\!=\!0,\nonumber \\ \end{aligned}$$

along with continuity of pressure and flux conditions on the interface between the vessel and the interior of the lobule. Implementing these conditions leads to an increase in the predicted lymph production of just under 1 %, while the proportionate change in the predicted overall blood flow was much smaller.

We also investigated the possible effect of alternative geometrical arrangements of the lobules. To do this, we considered a cuboid liver model consisting of lobules with a square cross section. We scaled the lobules so that their cross-sectional areas and the proportion of this area taken up by both portal tracts and central veins were preserved. We also ensured that the surface area and volume of the liver were preserved. With this model, we found that the predicted blood flow was reduced by about 24 %, while both the rate of lymph uptake and the total flux of fluid across the liver surface were about 12 % smaller than those in the case of the hexagonal lattice. The reduction in blood flow is to be expected; since the hexagonal arrangement has six portal tracts supplying each central vein, that arrangement has less resistance to flow than the square one.

Finally, we note that the results presented are based on the geometrical assumption that the lobules are orientated face-on to the surface of the liver, as opposed to end-on. To our knowledge, there is no indication about which case is more realistic. However, since our model suggests that variations in interstitial pressure are small, it is likely that such changes would have a relatively small effect on the predicted rate of lymph production.

## Concluding remarks

We have developed a new model of the microcirculation in the liver, which incorporates production and flow of lymph through the two major pathways: uptake by the lymphatic vessels and flow out of the liver through the surface into the peritoneal cavity or diaphragmatic space. We were able to estimate nearly all of the parameters from experimentally derived measurements, and we showed that the expected effect of geometrical variations in the lobules is relatively small. Even though the model is idealized, it provides useful information about lymph outflow and response to pathological states. The results of the model are consistent with physiological measurements.

The model is based on numerous simplifying assumptions on the geometry and mechanics. The most major geometrical assumptions are as follows:

- Cylindrical vessels (portal tracts and central veins) that are parallel to one another.
- Vessels arranged in a regular hexagonal lattice.
- With regard to the surface of the model liver, the vessels run parallel to it, the outermost lobules have the same cross-sectional area (see Fig. 1).

The main assumptions on the mechanics are as follows:

- Both sinusoids and interstitium can be modelled as a porous material obeying Darcy’s law.
- The flow is two-dimensional.
- Flux from sinusoids to interstitium is proportional to pressure difference (no oncotic effects).
- Lymph uptake has a linear relationship to pressure.
- Flux across liver surface is proportional to pressure difference.

The major weaknesses of the model are as follows:

- No account of effects of irregular geometry, especially near the surface.
- Various pressures are required as inputs to the model (pressures in portal tracts and central veins, base lymphatic pressure, and pressures in peritoneal cavity and diaphragmatic space). In practice, these pressures vary in response to blood flow conditions, and ideally, the model should be extended so that these are an output.
- Model cannot account for other orientations of lobules relative to the surface.

Many processes take place during liver disease, some of which are not fully understood. Gordon (2012) describes the current understanding of the main processes leading to the development of ascites. These commonly include fibrosis of the liver and active vasodilation, which are not accounted for in the model described in this paper. There is scope to extend our model to include some of these effects, and this should be undertaken in a future work.

Under normal physiological conditions, spatial variations in the interstitial pressure are much smaller than those in the sinusoidal pressure, while approximately 1.1 times as much fluid leaves the liver through the surface as that leaving via the lymphatic ducts.

If the portal pressure were increased, such as would occur in small-for-size liver syndrome, the model predicts significant increases both in the uptake by the lymphatic ducts and in the rate of fluid leaving through the surface of the liver. In order to develop this model into a predictive model for the severity of ascites, a model of the portal venous tree must be added so that pressures in the portal tracts can be related to those in the portal vein, and a model of the peritoneal cavity must be added so that the equilibrium pressure for a given flow rate of lymph from the liver can be found. The extended model would, for example, enable us to predict the consequences of different applied drainage rates.

## Figures and Tables

**Fig. 14:**
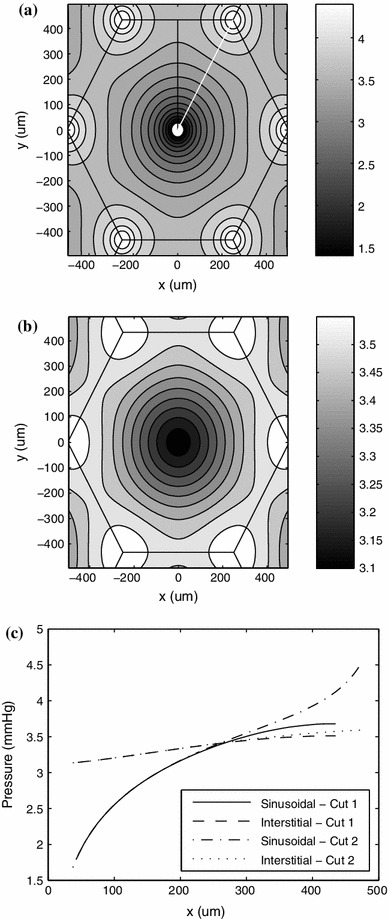
Analytic solution showing contours of **a** $p_S$, **b** $p_I$ in mmHg; the contours are spaced by **a** 0.2 mmHg, **b** 0.05 mmHg. **c** Pressures on Cuts 1 and 2 (shown in Fig. 3). The plots were produced with a lattice of about $3\times 10^6$ lobules, and this figure may be compared directly with those shown in Figs. 3 and 4

## Appendix A: Analytical model

Here we consider a simplified model of a lobule in the interior of the liver that we can solve analytically, which is based on the analytical model by Bonfiglio et al. (2010). As in that paper, we consider a regular lattice of lobules with portal tracts at the vertices and central veins along the axis and assume symmetry, but for simplicity, we treat these vessels as points in the plane. We denote by $\mathbf{x}_{PT,i}$ the location of the $i$th portal tract, and by $\mathbf{x}_{CV,j}$ the location of the $j$th central vein. In the case of point vessels, we cannot prescribe the sinusoidal and interstitial pressures there, so instead we prescribe the fluxes per unit length of the vessels. We assume that the flux of interstitial fluid from each of the portal tracts and central veins into the interstitium is zero, since these vessels have zero size in the model. The total length of all the lobules is the volume of the liver divided by the area of a lobule, $V_\mathrm{liv}/(3\sqrt{3}L_\mathsf{lob}^2/2)$, and thus, the total length of the portal tracts is twice this value. The volumetric flux of blood per unit length of portal tract from the portal tract into the sinusoids is assumed to be homogeneous throughout the model and equal to

14 $$\begin{aligned} q_{S,PT}&= \frac{Q_\mathrm{blood}}{2V_\mathrm{liv}/(3\sqrt{3}L_\mathsf{lob}^2/2)}\approx 6.3\times 10^{-9}\,\hbox {m}^2/\hbox {s}, \end{aligned}$$

where we used the flux found in the numerical calculations in the main part of the paper, rather than the physiological flux, for the purposes of comparison. We also assume that the volumetric fluxes from the sinusoids into the central vein per unit length are homogeneous and define these as

15 $$\begin{aligned} q_{S,CV}=2\left( 1-\gamma \right) q_{S,PT}, \end{aligned}$$

where $\gamma$ is the fraction of blood that is taken up by the lymphatic vessels, which is estimated in Appendix 6.5. Across the boundaries of the lobules, there is no flux in the sinusoids and free flow in the interstitial space.

The principle of superposition allows us to write the solutions of the governing Eqs. (6) and (7) as

16 $$\begin{aligned} p_S&= \sum _{\text {Portal tracts},i}p_{S,PT,i}+\sum _{\text {Central veins},j}p_{S,CV,j},\end{aligned}$$

17 $$\begin{aligned} p_I&= \sum _{\text {Portal tracts},i}p_{I,PT,i}+\sum _{\text {Central veins},j}p_{I,CV,j}, \end{aligned}$$

where $p_{S,PT,i}$ and $p_{I,PT,i}$ are the pressures in the sinusoids and the interstitium, respectively, in the case with a single portal tract (with the same boundary conditions) at $\mathbf{x}_{PT,i}$ and no central veins, and $p_{S,CV,j}$ and $p_{I,CV,j}$ are the corresponding pressures in the case with a single central vein at $\mathbf{x}_{CV,j}$.

Solving (6) for $p_I$, substituting into (7), and rearranging, we obtain a single governing equation for $p_S$:

18 $$\begin{aligned} \left( \nabla ^2-\lambda _1^2\right) \left( \nabla ^2-\lambda _2^2\right) \left( p_S-p_0\right) =0, \end{aligned}$$

where

19 $$\begin{aligned}&\lambda _{1,2}=\sqrt{\alpha \pm \sqrt{\alpha ^2- \frac{C_lC_f\mu _S\mu _I}{k_Sk_I}}},\end{aligned}$$

20 $$\begin{aligned}&\alpha =\frac{1}{2}\left( C_f\left( \frac{\mu _S}{k_S}+\frac{\mu _I}{k_I}\right) +\frac{C_l\mu _I}{k_I}\right) . \end{aligned}$$

Assuming that the pressures $p_{S,PT,i}$ and $p_{I,PT,i}$ are axisymmetric about the portal tract at $\mathbf{x}_{PT,i}$,

21 $$\begin{aligned} p_{S,PT,i}=C_1K_0\left( \lambda _1r_{PT,i}\right) +C_2K_0 \left( \lambda _2r_{PT,i}\right) , \end{aligned}$$

where $r_{PT,i}$ is the distance from $\mathbf{x}_{PT,i}$, $K_0$ is a modified Bessel function of the second kind, $C_1$ and $C_2$ are constants to be determined by applying boundary conditions, and we have used the fact that the pressures must decay far from the vessel to eliminate the modified Bessel functions $I_0$ that would also appear in the general solution. Hence,

22 $$\begin{aligned} p_{I,PT,i}&= p_{S,PT,i}-\frac{k_S}{C_f\mu _S}\nabla ^2p_{S,PT,i}\nonumber \\&= C_1\left( 1-\frac{k_S\lambda _1^2}{C_f\mu _S}\right) K_0\left( \lambda _1r_{PT,i}\right) \nonumber \\&\quad +\,C_2\left( 1-\frac{k_S\lambda _2^2}{C_f\mu _S}\right) K_0 \left( \lambda _2r_{PT,i}\right) . \end{aligned}$$

Similarly

23 $$\begin{aligned} p_{S,CV,j}&= C_3K_0\left( \lambda _1r_{CV,j}\right) +C_3K_0\left( \lambda _2r_{CV, j}\right) ,\end{aligned}$$

24 $$\begin{aligned} p_{I,CV,j}&= p_{S,CV,j}-\frac{k_S}{C_f\mu _S}\nabla ^2p_{S,CV,j}\nonumber \\&= C_3\left( 1-\frac{k_S\lambda _1^2}{C_f\mu _S}\right) K_0 \left( \lambda _1r_{CV,j}\right) \nonumber \\&\quad +\,C_4\left( 1-\frac{k_S\lambda _2^2}{C_f\mu _S}\right) K_0 \left( \lambda _2r_{CV,j}\right) , \end{aligned}$$

where $r_{CV,j}$ is the distance from the $j$th central vein and $C_3$ and $C_4$ are constants to be determined.

The volumetric flux per unit length out of the $i$th portal tract into the sinusoids equals

25 $$\begin{aligned} q_{S,PT}&= \lim _{\epsilon \rightarrow 0}\oint \limits _{r_{PT,i}=\epsilon }\mathbf{n}\cdot \mathbf{u}\,dl\nonumber \\&= \lim _{\epsilon \rightarrow 0}2\pi \epsilon \left( -\frac{k_S}{\mu _S}\right) \left( \lambda _1C_1K_0'\left( \lambda _1\epsilon \right) \right. \nonumber \\&\left. \quad +\,\lambda _2C_2K_0'\left( \lambda _2\epsilon \right) \right) \nonumber \\&= \frac{2\pi k_S}{\mu _S}\left( C_1+C_2\right) , \end{aligned}$$

where we used the fact that $\lim _{z\rightarrow 0}(zK_0'(z))=-1$, and, similarly, the flux per unit length from sinusoids to central vein is

26 $$\begin{aligned} q_{S,CV}=-\frac{2\pi k_S}{\mu _S}\left( C_3+C_4\right) , \end{aligned}$$

where the minus sign comes from the direction of the flux. The corresponding fluxes per unit length from the portal tracts into the interstitium and from the interstitium into the central vein both equal to zero, and hence,

27 $$\begin{aligned} 0&= \frac{2\pi k_I}{\mu _I}\left( \left( 1\!-\!\frac{k_S\lambda _1^2}{C_f\mu _S}\right) C_1 \!+\!\left( 1-\frac{k_S\lambda _2^2}{C_f\mu _S}\right) C_2\right) ,\qquad \end{aligned}$$

28 $$\begin{aligned} 0&= \frac{2\pi k_I}{\mu _I}\left( \left( 1\!-\!\frac{k_S\lambda _1^2}{C_f\mu _S}\right) C_3 \!+\!\left( 1-\frac{k_S\lambda _2^2}{C_f\mu _S}\right) C_4\right) .\qquad \end{aligned}$$

Solving these for the constants yields

29 $$\begin{aligned} C_1&= \frac{\mu _S(C_f\mu _S/k_S-\lambda _2^2)}{2\pi k_S(\lambda _1^2-\lambda _2^2)}q_{S,PT},\end{aligned}$$

30 $$\begin{aligned} C_2&= -\frac{\mu _S(C_f\mu _S/k_S-\lambda _1^2)}{2\pi k_S(\lambda _1^2-\lambda _2^2)}q_{S,PT},\end{aligned}$$

31 $$\begin{aligned} C_3&= -\frac{\mu _S(C_f\mu _S/k_S-\lambda _2^2)}{2\pi k_S(\lambda _1^2-\lambda _2^2)}q_{S,CV},\end{aligned}$$

32 $$\begin{aligned} C_4&= \frac{\mu _S(C_f\mu _S/k_S-\lambda _1^2)}{2\pi k_S(\lambda _1^2-\lambda _2^2)}q_{S,CV}. \end{aligned}$$

Substituting these expressions into (21)–(24), and then into (16) and (17), we obtain expressions for the pressures:

33 $$\begin{aligned} p_S&= \frac{\mu _S(C_f\mu _S/k_S-\lambda _2^2)}{2\pi k_S(\lambda _1^2-\lambda _2^2)}\nonumber \\&\times \quad \left( q_{S,PT}\sum _{\text {Portal tracts},i}K_0\left( \lambda _1r_{PT,i}\right) \right. \nonumber \\&\quad \left. -\,q_{S,CV}\sum _{\text {Central veins},j}K_0\left( \lambda _1r_{CV,j}\right) \right) \nonumber \\&\quad +\,\frac{\mu _S(C_f\mu _S/k_S-\lambda _1^2)}{2\pi k_S(\lambda _1^2-\lambda _2^2)}\nonumber \\&\times \quad \left( -q_{S,PT}\sum _{\text {Portal tracts},i}K_0\left( \lambda _2r_{PT,i}\right) \right. \nonumber \\&\quad \left. +\,q_{S,CV}\sum _{\text {Central veins},j}K_0\left( \lambda _2r_{CV,j}\right) \right) ,\end{aligned}$$

34 $$\begin{aligned} p_I&= \frac{(C_f\mu _S/k_S-\lambda _1^2)(C_f\mu _S/k_S-\lambda _2^2)}{2\pi C_f(\lambda _1^2-\lambda _2^2)}\nonumber \\&\times \quad \left( q_{S,PT}\sum _{\text {Portal tracts},i}\left( K_0\left( \lambda _1r_{PT,i}\right) \right. \right. \nonumber \\&\quad \left. -\,K_0\left( \lambda _2r_{PT,i}\right) \right) \!+\!q_{S,CV}\sum _{\text {Central veins},j}\!\!\left( -K_0\left( \lambda _1r_{CV,j}\right) \right. \nonumber \\&\quad \left. \left. +\,K_0\left( \lambda _2r_{CV,j}\right) \right) \right) . \end{aligned}$$

We note that, by symmetry, these solutions automatically satisfy the conditions on the boundaries between lobules. The solution is calculated using Matlab and is shown in Fig. 14.

The analytical solution has the advantage with respect to the numerical one presented in the main part of the paper that it allows us to resolve better the details of the pressure near portal tracts and central veins, which is where changes are more significant.

## Appendix B: Estimation of model parameters from experiments

### Surface area of the liver

Negrini et al. (1990) measured the surface area of five rabbit livers and found them to be $240\,\pm \,13 \hbox {cm}^2$. We use the data from Boxenbaum (1980) to scale this up to the human: Typical body masses for rabbit and human are 2.88 and 62.8 kg, while liver masses as a fraction of body mass are 4.78 and 2.42 %, respectively. Estimating that areas scale as the two-thirds of the power of volumes gives a surface area of

35 $$\begin{aligned} A_\mathrm{liv}=\left( \frac{0.0242\times 62.8}{0.0478\times 2.88}\right) ^{2/3}240 =1190\,\hbox {cm}^2. \end{aligned}$$

### Interstitial permeability

We were unable to find experimental data on the value of the model parameter $k_I$, so here we develop a model to estimate its value. Interstitial fluid is contained in the space of Disse and also in the gaps between cells of the liver. The space of Disse surrounds the sinusoids and contains the vast majority of the interstitial fluid and also, due to its relatively large width, offers much less resistance to fluid flow than the gaps between cells. Thus, we assume that the permeability of the interstitial space as a whole is dominated by the permeability of the network of vessels comprising the space of Disse. We use a simplified model of the geometry of the space of Disse and the Kozeny–Carman relationship to estimate the permeability. This relationship states that the permeability equals $\phi ^3/(cS^2)$, where $\phi$ is porosity, $S$ is the specific surface, defined as wet surface area per unit total volume, and $c$ is the Kozeny constant.

We treat the sinusoids as cylinders of diameter $D_\mathrm{sin}=10\,\upmu$m (Burt et al. 2006) surrounded by an annular region of width $D_{SD}=0.5$ $\upmu$m (estimated from a diagram in Burt et al. (2006)) representing the space of Disse. Assuming that the Kozeny constants of the sinusoids and interstitium are equal gives the relationship:

36 $$\begin{aligned} k_I=\left( \frac{S_S}{S_I}\right) ^2\left( \frac{\phi _I}{\phi _S}\right) ^3k_S, \end{aligned}$$

where $\phi _S$ and $\phi _I$ are the porosities of the sinusoids and interstitium, respectively, and $S_S$ and $S_I$ are the corresponding specific surfaces. The ratio of porosities is estimated as the ratio of cross-sectional areas, that is,

$$\begin{aligned} \frac{\phi _I}{\phi _S}\approx \frac{(\pi D_\mathrm{sin}D_{SD})}{(\pi D_\mathrm{sin}^2/4)}=\frac{4D_{SD}}{D_\mathrm{sin}}. \end{aligned}$$

The wet surface area of the space of Disse is approximately twice that of the sinusoid, meaning that $S_I/S_S\approx 2$. Hence,

37 $$\begin{aligned} k_I\approx \frac{1}{4}\left( \frac{4D_{SD}}{D_\mathrm{sin}}\right) ^3k_S=\frac{16D_{SD}^3}{D_\mathrm{sin}^3}k_S=0.002k_S. \end{aligned}$$

### Hepatic filtration

Greenway et al. (1969) performed experiments on the livers of anesthetized cats in which they controlled the hepatic venous pressure and measured arterial and portal pressure, liver volume, and blood flow through the liver in order to determine the filtration coefficient, which is the volumetric flow from the sinusoids to the interstitium per unit mechanical pressure difference between the sinusoids and the interstitium and per unit liver mass. They found that the flow rate was $F=0.30\pm 0.03$ ml/min per mmHg pressure difference between sinusoids and interstitium per 100 g of liver tissue. In our model, we define the hepatic filtration coefficient, $C_f$, as the volumetric rate of blood flow from sinusoids to interstitium per unit pressure drop between sinusoids and interstitium per unit volume of tissue. This is given by

38 $$\begin{aligned} C_f&= \rho _t F =1060\times \left( 0.30\times \frac{1}{10^6\times 60}\times 10\right) \nonumber \\&= 5.3\times 10^{-5}\text {/(mmHg s)}, \end{aligned}$$

where $\rho _t=1{,}060\,\hbox {kg/m}^3$ is the density of liver tissue (Kotiluoto and Auterinen 2004).

### Conductance of the lymphatic ducts

Elk et al. (1988) performed experiments on anesthetized dogs weighing 20–30 kg to determine the flow rate into the lymphatic vessels as a function of interstitial pressure. They found that the volumetric flux of lymph leaving the liver equaled $\max (p_I-p_0,0)/R_l$, where $R_l=0.056\,\hbox {cmH}_2$O min/$\mu \hbox {l}=0.056\times (10/13.6) \times (60\times 10^9)=2.5\times 10^9\, \hbox {mmHg s/m}^3$ is the resistance of the ducts. Boxenbaum (1980) gives the typical mass of the dog liver as 2.91 % of body weight, and, taking the typical mass of the dogs in the experiment as 25 kg, this gives the volume flux per unit volume of liver tissue as $C_l\max (p_I-p_0,0)$, where

39 $$\begin{aligned} C_l&= \frac{1}{R_l}\frac{\rho _t}{0.0291\times 25}=\frac{1}{2.5\times 10^9} \frac{1060}{0.0291\times 25}\nonumber \\&= 5.9\times 10^{-7}\text {/(mmHg s)}, \end{aligned}$$

where $\rho _t=1{,}060\,\hbox {kg/m}^3$ is the density of liver tissue (Kotiluoto and Auterinen 2004).

### Fraction of blood that is taken up by the lymphatic vessels

Laine et al. (1979) measured the typical outflow of lymph via the lymphatic vessels (not the surface) from the livers of anesthetized dogs, finding it to be $3.5\pm 1.19$ ml/h. We scale this up to the typical flow rate for humans by multiplying by the ratio of liver mass of humans to that of dogs. The weight of the animals was recorded as at least 17 kg (here we take it as 17 kg), 62.8 kg is used as a typical human body mass, and the liver masses are 2.91 and 2.42 % of the body masses for dogs and humans, respectively (Boxenbaum 1980). Thus, the flux of lymph uptake from a human liver is estimated as

40 $$\begin{aligned} Q_L&= 3.5\times \frac{62.8\times 0.0242}{17\times 0.0291} =10.75\,\text {ml/h}\nonumber \\&= 3.0\times 10^{-9}\,\hbox {m}^3/\hbox {s}. \end{aligned}$$

The flux of blood through the liver is 1,717 ml/min  (Wynne et al. 1989 see also Table 1), and thus, we estimate that under normal physiological conditions,

41 $$\begin{aligned} \gamma =\frac{Q_L}{Q_\mathrm{blood}}=1.0\times 10^{-4}. \end{aligned}$$
